# Diagnostic and Therapeutic Dilemmas in Recurrent Pulmonary Alveolar Proteinosis After Bilateral Lung Transplantation

**DOI:** 10.1002/rcr2.70653

**Published:** 2026-06-12

**Authors:** Omar Lopez, Deven Gupta, Shreya Podder, Ajay Wagh

**Affiliations:** ^1^ Section of General Internal Medicine University of Chicago Chicago Illinois USA; ^2^ Department of Pathology University of Chicago Chicago Illinois USA; ^3^ Section of Pulmonary and Critical Care University of Chicago Chicago Illinois USA

**Keywords:** GM‐CSF antibodies, lung transplantation, opportunistic infections, recurrent pulmonary alveolar proteinosis, whole‐lung lavage

## Abstract

Pulmonary alveolar proteinosis (PAP) is a rare granulocyte–macrophage colony‐stimulating factor (GM‐CSF) dependent lung disease treated primarily with whole‐lung lavage (WLL); recurrence after lung transplantation is uncommon and poorly characterised. We present a 73‐year‐old Hispanic man with PAP who developed recurrent disease more than 3 years after bilateral lung transplantation. High‐resolution CT showed evolving ground‐glass opacities and crazy‐paving, and anti–GM‐CSF antibodies were positive, yet repeated bronchoalveolar lavage and transbronchial biopsies were initially PAS‐negative. Concomitant *Mycobacterium avium* complex, Aspergillus and Nocardia infections mimicked recurrent PAP and coincided with an atypical shift in spirometry from supranormal airflow (FEV_1_ 110% predicted; FEV_1_/FVC 91%) to obstruction (FEV_1_ 99% predicted; FEV_1_/FVC 68%). Ultimately, repeat lavage revealed PAS‐positive lipoproteinaceous material, and treatment with inhaled sargramostim plus two sequential WLLs produced marked radiographic clearance, symptomatic relief and partial spirometric recovery. Our case highlights how multimodal, longitudinal clinical surveillance remains essential in lung transplant recipients.

## Graphical abstract

Pulmonary alveolar proteinosis (PAP) is an ultra‐rare lung disease that can recur even years after bilateral lung transplantation, as in this 73‐year‐old man whose diagnosis was delayed by PAS‐negative BAL and overlapping opportunistic infections (Nocardia, MAC, Aspergillus). Despite initially normal post‐transplant spirometry, he developed progressive dyspnea, new ground‐glass and crazy‐paving opacities and mild airflow obstruction, ultimately leading to confirmation of recurrent autoimmune PAP on whole‐lung lavage (WLL). Treatment with inhaled GM‐CSF (sargramostim) plus sequential WLL produced marked symptomatic, radiologic and spirometric improvement, underscoring the need for multimodal, longitudinal surveillance and the continued effectiveness of standard PAP‐directed therapies in the transplanted lung.

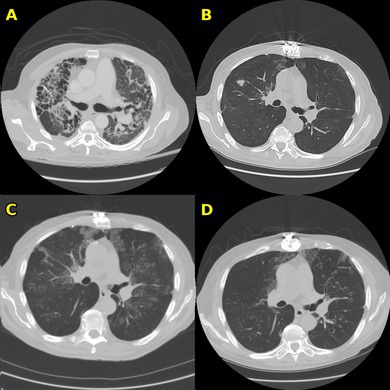

## 1 Introduction

Pulmonary alveolar proteinosis (PAP) is a rare diffuse lung disease characterised by intra‐alveolar accumulation of lipoproteinaceous surfactant due to impaired clearance by alveolar macrophages or disrupted granulocyte–macrophage colony‐stimulating factor (GM‐CSF) signalling. PAP usually presents with progressive exertional dyspnea and non‐productive cough, but these non‐specific symptoms are often attributed to more common disorders [1, 2, 3].

Diagnosis of PAP is multimodal and consists of high‐resolution computed tomography (HRCT), bronchoalveolar lavage (BAL) and serology: HRCT often demonstrates a ‘crazy‐paving’ pattern, BAL may show periodic acid–Schiff (PAS)–positive lipoproteinaceous material, and anti–GM‐CSF antibodies are highly specific for autoimmune PAP [1, 2, 3].

Standard therapy for symptomatic PAP is whole‐lung lavage (WLL), with GM‐CSF–based therapies, including inhaled sargramostim, which are used as adjuncts in autoimmune disease. Lung transplantation is reserved for refractory PAP and is rarely required; recurrence after transplantation is considered even rarer and remains poorly characterised [4, 5].

## 2 Case Report

A 73‐year‐old Hispanic man was diagnosed with PAP in 2001 by video‐assisted thoracoscopic lung biopsy after an incidental abnormal chest x‐ray. Anti–GM‐CSF antibodies were first documented in 2002, with a negative result in 2017. Comorbidities included hypertension, hypothyroidism, obstructive sleep apnea with excellent CPAP adherence, chronic kidney disease stage 3b, atrial fibrillation and type 2 diabetes mellitus. He had occupational exposure as an electrician when he worked for coal and steel plants with concern for coal/silica dust inhalation, but no tobacco, alcohol or illicit drug use.

For a decade after diagnosis he used only albuterol as needed. He experienced slowly progressive, exertional dyspnea and ultimately underwent two WLL, two courses of pulmonary rehabilitation, and 6 months of inhaled sargramostim without sustained benefit. In 2020 he experienced marked decline after a bout of community‐acquired pneumonia followed by *Mycobacterium avium* –intracellulare infection. Shortly after he was listed for transplantation and underwent bilateral sequential lung transplantation in November 2021.

Post‐transplant prophylaxis included itraconazole, valganciclovir and sequential Pneumocystis coverage (trimethoprim–sulfamethoxazole, dapsone, atovaquone, lastly aerosolized pentamidine), along with belatacept in the setting of Epstein–Barr virus seropositivity. Maintenance immunosuppression consisted of prednisone and tacrolimus, with azathioprine later switched to mycophenolate mofetil for renal protection.

Initially, he had excellent functional recovery with near‐normal spirometry and reassuring surveillance bronchoscopies as can be noted in Tables 1 and 2 respectively. Early bronchoscopies identified isolated *Proteus mirabilis* and low‐level Serratia colonisation, importantly without rejection. Mild dyspnea in 2022 prompted chest imaging that showed new opacities, and anti–GM‐CSF antibodies were again detected, but titers were not quantified. Given overall stability, close observation with interval imaging was planned.

**TABLE 1:** Representative longitudinal spirometry trend from pre‐transplant decline to post‐transplant stability.

Value	12/1/2020	3/23/2021	6/16/2021	6/22/2021	8/25/2021	8/1/2023	2/20/2024	5/16/2025
FVC (L) PRED	3.98	3.95	3.95	3.95	2.47	3.9	3.9	3.85
FVC (L)	3.14	2.5	2.46	2.3	2.38	3.95	3.77	3.73
FVC (L) %PRED	78	63	62	58	96	101	96	97
FEV1 (L) PRED	2.73	2.7	2.7	2.7	1.78	2.63	2.63	2.56
FEV1 (L)	2.48	1.99	1.73	1.89	1.81	2.92	2.97	2.66
FEV1 (L) %PRED	90	73	64	70	102	110	112	103
FEV1/FVC PRED (%)	69	68	68	68	72	67	67	67
FEV1/FVC (%)	79	80	70	82	76	74	79	71
FEV1/FVC %PRED	115	116	103	120	106	109	116	107
FEF25–75 PRED (L/s)	2.6	2.56	2.56	2.56	2.09	2.47	2.47	2.37
FEF25–75 (L/s)	2.3	1.81	1.43	1.96	1.53	2.21	2.6	1.71
FEF25%–75% PRED	88	70	56	76	73	89	105	72

**TABLE 2:** Post‐transplant bronchoscopies with histology and microbiology.

Date	Time post‐TX	Indication	Histology/cytology	BAL/biopsy microbiology	Management
01/14/2022	≈1 month	Routine surveillance	A0BX^a^	*Proteus mirabilis*	Inhaled tobramycin × 28 days
02/25/2022	≈3 months	Routine surveillance	Left lower lobe AXB0; lingula A0B0	Cultures negative	No new therapy
05/05/2022	≈6 months	Routine surveillance	Right lower lobe A0B0; RML A0BX	600 CFU serratia	Observed as colonisation; no specific therapy
08/01/2022	≈9 months	Surveillance (during tacrolimus adjustment)	A0B0	No significant growth	No new therapy
10/12/2022	1 year	Annual surveillance	A0B0; cytology negative (PAS‐negative)^b^	BAL cultures negative	No new therapy; continued routine follow‐up
11/16/2023	≈2 years	RUL 13 × 14 mm lobulated, spiculated nodule noted on CT from 10/26/2023	Biopsy/FNA: non‐necrotizing granuloma; GMS/AFB negative	*Nocardia abscessus*	Prolonged combination IV/PO therapy (including TMP‐SMX, ceftriaxone, amoxicillin‐clavulanate, doxycycline), then lifelong doxycycline secondary prophylaxis
08/14/2024	≈2.5 years	Worsening dyspnea; scattered GGOs/crazy‐paving on CT	A0B0; cryobiopsy PAS‐negative	No dominant new pathogen; background of prior Nocardia	Initiated inhaled sargramostim; planning for whole‐lung lavage in context of suspected recurrent PAP
02/11/2025With WLL	≈3.1 years	Progressive dyspnea, GGOs/crazy‐paving; pre−/peri‐WLL evaluation	A0B0; abundant PAS‐positive lipoproteinaceous material	BAL: Aspergillus sydowii and MAC (MAI)^c^	Antifungal therapy with voriconazole then posaconazole; initiation of triple MAC therapy (rifabutin, ethambutol, azithromycin) with planned 12‐month course after sputum conversion; sequential whole‐lung lavages and continued inhaled sargramostim

^a^ Biopsy grades reported using ISHLT lung allograft rejection criteria: A, acute cellular rejection; B, airway inflammation (lymphocytic bronchiolitis); 0, absent; higher numbers, increasing severity; X, indeterminate/insufficient tissue.

^b^ No samples were retrospectively reviewed.

^c^ Quantitative burden of microbiology pathogens was not reported.

In October 2023, HRCT revealed a new cavitary lesion in the right upper lobe, and bronchoscopy confirmed Nocardia infection, which was treated with prolonged multidrug therapy and transition to lifelong doxycycline prophylaxis. During subsequent follow‐up, additional opportunistic infections with *Mycobacterium avium* complex and Aspergillus species were documented. Donor‐specific HLA antibodies remained negative.

Over the following year, exertional dyspnea progressed and a new supplemental oxygen requirement emerged. HRCT demonstrated scattered ground‐glass opacities and a crazy‐paving pattern concerning for recurrent PAP versus infection, rejection or chronic lung allograft dysfunction. Serial spirometry showed preservation of forced vital capacity and initially supranormal airflow, followed by a shift to a mildly obstructive pattern.

Bronchoscopy with biopsies in August 2024 revealed no histologic evidence of PAP, and PAS staining of BAL and tissue was negative. Despite this, symptoms and imaging abnormalities progressed. In early 2025, given persistent clinical concern and positive anti–GM‐CSF antibodies, inhaled sargramostim was initiated and WLL was performed in February 2025. Lavage fluid demonstrated abundant PAS‐positive lipoproteinaceous material, confirming recurrent PAP as can be seen in Figure 1. A second WLL in April 2025 resulted in marked symptomatic improvement, radiologic clearing of ground‐glass and crazy‐paving changes, and partial spirometric recovery as seen in Figure 2.

**FIGURE 1:**
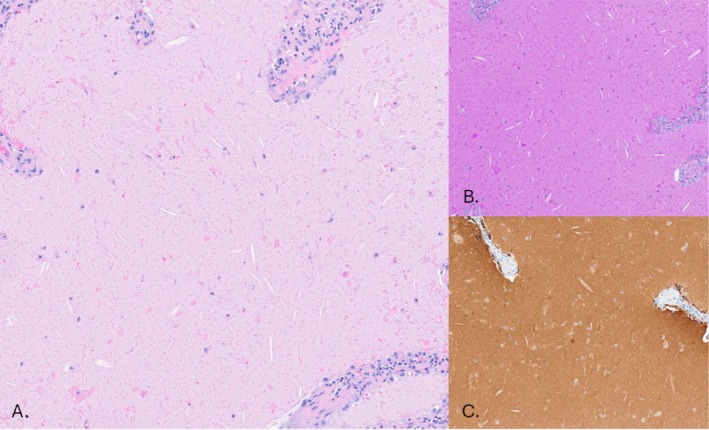
Surgically explanted native right lung with pulmonary alveolar proteinosis (PAP). (A) H&E‐stained section at 10× magnification demonstrates finely granular, acellular eosinophilic material and cholesterol clefts within alveolar airspaces. (B) PAS‐stained section at 10× magnification demonstrates strong staining of the acellular, crystalline structures. (C) Napsin‐A‐strained section at 10× magnification demonstrates strong and homogenous staining.

**FIGURE 2:**
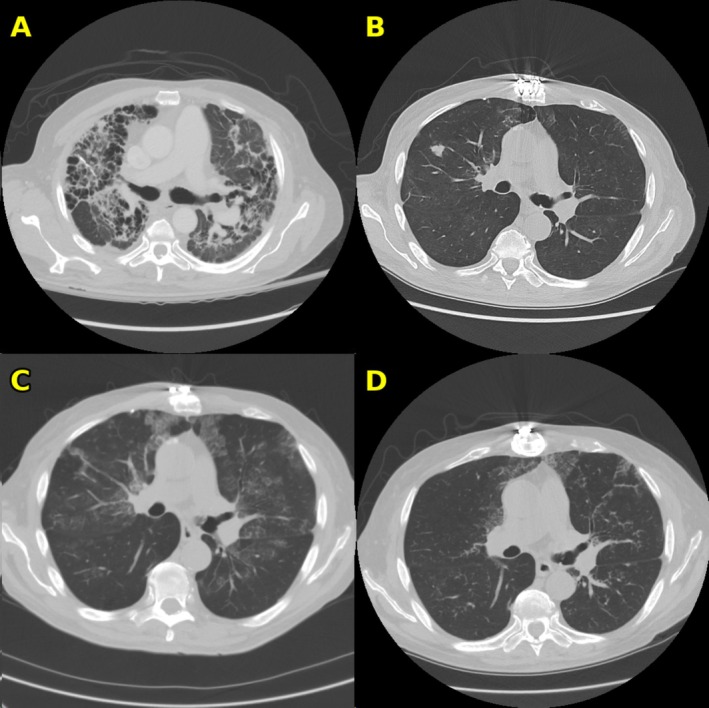
Serial CT chest imaging before and after bilateral lung transplantation, nocardial infection, PAP recurrence and whole‐lung lavage (WLL). (A) CT chest from August 24, 2021, prior to bilateral lung transplant. (B) CT chest from September 24, 2023, after bilateral lung transplant when the nocardia lesion was identified. (C) CT chest from October 14, 2024, demonstrating worsening pulmonary alveolar proteinosis. (D) CT chest from March 16th, 2025, after the first WLL.

## 3 Discussion

This case demonstrates that recurrent PAP can manifest more than 3 years after transplantation and may remain obscure despite strongly suggestive clinical and radiologic features. Repeated PAS‐negative BAL and biopsies initially delayed diagnosis, emphasising that a single negative study does not exclude recurrence when HRCT shows evolving ground‐glass opacities and crazy‐paving and the clinical trajectory is consistent with PAP [2, 3]. Longitudinal, multimodal reassessment integrating imaging, serology, bronchoscopy and pulmonary‐function testing is crucial in such patients [1, 2, 3].

The presence of concomitant and co‐incidental opportunistic infections—Nocardia, *Mycobacterium avium* complex and Aspergillus—further complicated interpretation [4, 5]. These pathogens can produce diffuse opacities and cavitary lesions that overlap radiographically with PAP, obscure patterns of rejection or chronic lung allograft dysfunction and alter spirometric patterns [4, 5]. As we saw in this patient, the evolution from supranormal post‐transplant airflow to mild obstruction was atypical for isolated PAP and could reasonably be attributed to infection or chronic lung allograft dysfunction. Anti–GM‐CSF antibodies, negative before transplant but positive when recurrence was suspected, also had to be interpreted without quantitative values to correlate burden and in the context of active infection as well as heightened immune dysregulation [1, 2, 3]. Collectively, these factors argue for a parallel and critical evaluation strategy in prior‐PAP transplant recipients with new diffuse opacities and functional decline.

The delayed recurrence of autoimmune PAP more than 3 years after transplantation may reflect progressive repopulation of the allograft by recipient‐derived alveolar macrophages, with re‐emergence of impaired GM‐CSF–dependent surfactant clearance. However, neither macrophage chimerism nor donor‐specific GM‐CSF signalling status was assessed in this patient. Therapeutically, this case shows that recurrent autoimmune PAP in the transplanted lung remains highly responsive to conventional PAP‐directed therapy [1, 2, 3]. Inhaled sargramostim combined with two sequential WLL produced rapid symptomatic, radiologic and physiologic improvement. The apparent difference in therapeutic response before and after transplantation should be interpreted cautiously as differences in disease burden, parenchymal reserve, timing of therapy and concomitant infection may have contributed to the observed post‐transplant response; however, the mechanism cannot be established from a single case. These findings support the concept that lung transplantation does not correct underlying GM‐CSF–dependent defect and that the allograft remains amenable to WLL and GM‐CSF augmentation when PAP recurs [1, 2, 3]. Finally, the patient's course underscores a potential value of structured, longer‐term surveillance by using periodic HRCT, serial pulmonary function testing and repeat bronchoscopy with BAL at least for 3 years in transplant recipients.

## Author Contributions

Omar Lopez wrote the original draft of the manuscript. All authors reviewed the work critically for important intellectual content and approved the final draft of the manuscript for publication.

## Funding

The authors have nothing to report.

## Consent

The authors declare that written informed consent was obtained for the publication of this manuscript and accompanying images and attest that the form used to obtain consent from the patient complies with the Journal requirements as outlined in the author guidelines.

## Conflicts of Interest

The authors declare no conflicts of interest.

## Data Availability

The data that support the findings of this study are available on request from the corresponding author. The data are not publicly available due to privacy or ethical restrictions.
